# Minimally Invasive Gastrointestinal Surgery: A Review

**DOI:** 10.7759/cureus.48864

**Published:** 2023-11-15

**Authors:** Sejal S Singh, Raju K Shinde

**Affiliations:** 1 Surgery, Jawaharlal Nehru Medical College, Datta Meghe Institute of Higher Education and Research, Wardha, IND; 2 General Surgery, Jawaharlal Nehru Medical College, Datta Meghe Institute of Higher Education and Research, Wardha, IND

**Keywords:** small bowel, pancreas, colorectal, minimally invasive laparoscopy, gi surgery

## Abstract

Minimally invasive surgery uses several procedures with fewer side effects (bleeding, infections, etc.), a shorter hospital stay, and less discomfort following minimally invasive surgery. Laparoscopy was one of the first forms of minimally invasive surgery. It involves doing surgery while using tiny cameras through one or more small incisions, surgical tools along with tubes. Robotic surgery is another kind of minimally invasive procedure. Along with supporting accurate, flexible, and regulated surgical procedures, it provides the physician with a three-dimensional, enlarged view of the operative site. Minimally invasive surgery continues to advance, making it an advantage for patients with a variety of illnesses. Nowadays, many surgeons prefer it to traditional surgery, which frequently necessitates a longer hospital stay and requires larger incisions. Since then, numerous surgical specialties have greatly increased their use of minimally invasive surgery.

A minimally invasive procedure is preferred for the majority of patients who require gastrointestinal surgery. Minimally invasive gastrointestinal procedures are just as successful as open procedures and, in some situations, may result in more effective outcomes. While recovery from open surgeries frequently takes five to ten days in the hospital, minimally invasive surgeries are less painful for patients and hasten recovery. It is safe from the perspective of the patient and has a lower postoperative mortality rate. This procedure involves a learning curve among surgeons.

## Introduction and background

The first laparoscopic removal of the gall bladder was performed by Philippe Mouret in 1987, bringing in a new era for surgery. Operative surgery has been revolutionized by minimally invasive procedures, which are now utilized in the majority of surgical subspecialties like laparoscopy, arthroscopy, gastroscopy, hysteroscopy, and sigmoidoscopy. However, laparoscopic surgery, in particular, has downsides. The restricted degrees of freedom on inflexible surgical instruments reduces surgical competence. Additionally, the surgeon uses lengthy instruments to keep his hands away from the surgical area. Computer and robotic surgical advancements have looked at novel ways to overcome restrictions, and technology is gradually being used into clinical practice. Computer systems make preoperative planning and instrument positioning easier, while robotic surgical arms increase surgical method precision [1]. For the first time, robot-assisted surgery was performed in 1988 by Davies et al. This technique resulted in the creation of Probot, a second-generation robot made exclusively for transurethral prostate excision [2,3]. The indications in emergency scenarios because of technological challenges are the most disputed aspects in minimally invasive procedures. These technological issues have been resolved for a variety of conditions, including acute appendicitis and perforated peptic ulcers, for which a laparoscopic approach has gained wide acceptance. However, endoscopic procedures now have more indications, pushing surgical treatments to the back of the line. Examples include cholangitis and pancreatic abscess drainage. By using an endoluminal method, these disorders can be treated without the requirement for laparoscopic development. However, new tools and technology might enable the laparoscopic method to be used for a wider range of prospective treatments [4]. Following are a few conditions where laparoscopic surgeries are used: (1) esophageal perforation: In comparison to conventional surgery, recent developments in minimally invasive procedures have led to a reduction in rates of relapse, open surgical interventions, complications and morbidity, (2) perforated peptic ulcer: laparoscopic closure of perforated ulcers has been reported to be feasible and safe, the advantages of using this minimally invasive approach are numerous, it is extremely simple to do laparoscopic simple patch closure of the perforation, and it appears to be an effective treatment, (3) laparoscopic cystogastrostomy: using laparoscopic methods, a fistula is made between the cystic cavity and the stomach by removing necrotic tissue and draining it, (4) retroperitoneal laparoscopic debridement: in cases of severe acute pancreatitis, both flanks may be utilized to drain infected necrosis, depending on where the necrosis is located, (5) acute small bowel obstruction (ASBO): a common surgical emergency called small bowel obstruction (SBO) is frequently caused by postoperative adhesions, majority of patients with adhesive SBO recover without surgery, although a significant number of individuals require urgent surgery, the most effective treatment for adhesive SBO has long been open surgery, for many elective conditions, laparoscopy is currently the first line of treatment, (6) Meckel’s diverticulum: laparoscopic diverticulectomy can speed up patient discharge and is as safe and successful as open surgery [4-7]. The indications for therapeutic endoscopy have increased as a result of improvements in the design of flexible endoscopes and endoscopic equipment and the rise in demand for minimally invasive surgeries [8].

## Review

Methodology

**Figure 1:**
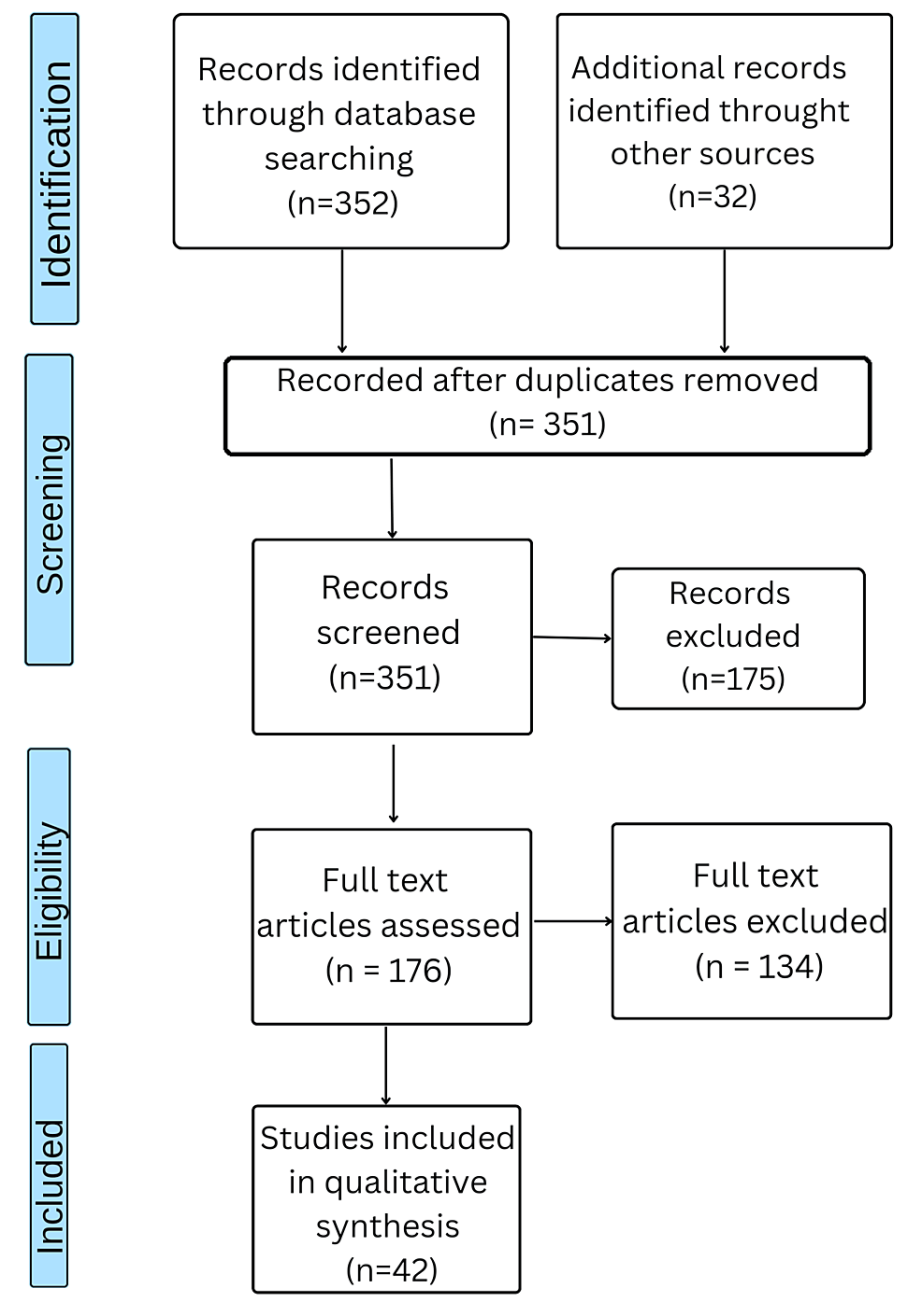
The selection process of articles used in this study. Adapted from Preferred Reporting Items for Systemic Reviews and Meta-Analyses (PRISMA).

To find appropriate research for this review article, a thorough literature search was carried out. A search was conducted using terms relating to small bowel, pancreas, colorectal, minimally invasive surgery (MIS), and GI surgery in electronic databases such as PubMed. Only articles written in English were included in the search. Further relevant research was found by consulting with specialists in the subject and looking through the reference lists of the identified articles. All papers on minimally invasive gastrointestinal surgery were eligible for inclusion, regardless of study style. Research that was not specifically related to gastrointestinal surgery or that had little bearing on minimally invasive surgery was disregarded. Title and abstract reviews were conducted as part of the screening process, and then the complete text was assessed to make sure the work was eligible for inclusion. By using these stringent selection criteria, the review article incorporates research that meets our eligibility requirements and offers trustworthy data on minimally invasive surgery. The method utilized to choose the papers for our study is shown in Figure 1.

A laparoscopic approach to various conditions is a proven benefit for patients, as it requires a small recovery period and a few days of hospital stay and also reduces the medications for recovery, along with a better cosmetic outcome in comparison to an open surgery procedure. The technique involves a camera that gives a visual display of the organ operating on it and instruments designed for the procedures, which are being guided with the help of an endoscope or laparoscope. There is always a learning curve in laparoscopic techniques, as the technologies are advancing with time and so are the methods of surgery, opening up a wide area for numerous procedures being done via this method. It’s a widely used procedure worldwide. With advancing methods, laparoscopic surgery is slowly shifting from elective procedures to emergency surgeries as well. Following are the various conditions of the gastrointestinal system where a laparoscopic approach is used.

Laparoscopic method in upper gastrointestinal conditions

*Esophageal Perforation* 

Esophageal perforation most commonly results from iatrogenic instrumentation of the esophagus. Recent advancements in endoscopic and minimally invasive techniques have resulted in lower rates of morbidity and mortality when compared to conventional surgery [5]. If there is an uncontrolled leak into the chest, there are several treatment options available, including laparoscopic or thoracoscopic emptying and feeding jejunostomy combined with an esophageal tube; if there is an uncontrolled leak into the abdomen within 24 hours, there are several options for treatment, including minimally invasive surgery (MIS) a gastric wrap and jejunostomy; and if there is an uncontrolled leak into the abdomen longer than 24 hours, there are several options for treatment, including a feeding jejunostomy and stent plus laparoscopic drainage [9]. 

*Perforated Peptic Ulcer* 

In the treatment of perforated peptic ulcers, the therapeutic goals are to close the breach in the GI tract and to address any peritoneal contamination. Higher perforation rates are present in the gastric body (18%), pyloric area (20%), and duodenal bulb (62%) [10]. Numerous significant studies have already been reported on the efficacy and safety of laparoscopic closure of perforated ulcers. This minimally invasive procedure has a lot of potential advantages. It is relatively simple to do laparoscopic simple patch closure of the perforation, and it appears to be an efficient treatment [6].

Upper GI Carcinoma

In 2012, esophageal cancer (EC) was the eighth most prevalent cancer worldwide and the sixth most common cause of cancer mortality [11]. The late 1980s saw the introduction of MIS, which has been defined as minimal access employing a laparoscope with exhaustion of CO_2_. In an effort to enhance postoperative outcomes, MIS has been utilized more frequently for upper GI malignancies, despite the fact that its effect on postoperative inflammatory response is yet unknown [12]. The surgical procedure of choice for esophageal cancer is increasingly being minimally invasive esophagectomy. By utilizing the advantages of lower mortality associated with minimally invasive methods, this technique aims to decrease the rate of respiratory issues related to thoracotomy [13]. 

*Esophageal* *Achalasia*

The most typical motor disorder and the second most typical functional disorder of the esophagus are both esophageal achalasia. The goal of current achalasia treatment is to totally remove the lower esophageal sphincter's inability to fully relax after swallowing. Endoscopic pneumatic dilation has almost been surpassed as the primary therapeutic strategy by the advancement of laparoscopic surgery [14]. Laparoscopy and thoracoscopy were both employed in the initial days of minimally invasive surgery while performing a Heller myotomy. But it quickly became apparent that laparoscopy had a number of built-in benefits, such as improved imaging of the junction between the esophagus and the stomach, single lumen intubation via the endotracheal tube, the ability to incorporate an anti-reflux procedure, and a reduced length of hospital stay [15].

Laparoscopic method for hepatobiliary condition

Acute Cholecystitis

Acute cholecystitis is the third most common cause of severe abdominal pain in emergency rooms, accounting for twenty to thirty percent of cases with biliary colic, the most prevalent biliary tree's infection. Recently, there have been changes in how acute cholecystitis is treated. Although the laparoscopic technique has revolutionized the treatment of biliary illnesses, laparoscopic cholecystectomy is not the preferred procedure for the management of gallbladder lesions or acute cholecystitis [16]. Table 1 depicts the treatment of cholecystitis based on the grades of severity.

**Table 1:** Acute cholecystitis treatment based on severity. Source: [4].

S. no.	Management	Grade
1	The preferred course of treatment is sufficient support of the organ along with supervision, drainage of bile, or an urgent removal of the gall bladder. The preferred course of treatment for choleperitoneum following gallbladder perforation is immediate laparoscopic cholecystectomy.	Severe
2	It is preferable to have a laparoscopic cholecystectomy performed by skilled doctors. The best course of action in cases of severe inflammation is an emergency laparoscopic cholecystectomy.	Moderate
3	Initial laparoscopic removal of the gallbladder is the preferred course of action. For patients at high risk, conservative treatment is advised.	Mild

Hepatic Abscess

The mortality rate for hepatic abscesses ranges from 6 to 14% [17]. Early intravenous antibiotic therapy initiation is necessary for initial care. The most commonly used method, percutaneous aspiration, has a success rate of 85%-95% with low morbidity and death. Age fifty-five years, abscess of approximately 5 cm, involving both the lobes of the liver, and illness lasting for seven days or more are risk factors for aspiration [18]. When aspiration through the skin is risky or unlikely to succeed due to a severally distributed abscess, biliary communication, or excessive levels of urea, creatinine, or total bilirubin, surgery is advised as a treatment option for biliary abscesses, intra-abdominal collections brought on by surgery, or in instances when the patient has biliary abscesses [19]. 

*Liver Transplantation* 

For individuals with chronic liver disease, transplanting the liver can save lives. While keeping recipients' access to liver transplantation intact, lowering the rates of mortality and morbidity is also a target. Due to issues regarding hemostasis, the safety of the donor, and the quality of the graft, laparoscopy has taken longer to advance for living donor hepatectomies than it has for donor nephrectomy. For left lateral sectionectomy, a strict minimally invasive method has become the standard procedure. A number of hospitals with decades of experience using living donors and laparoscopic surgery have reported using a fully minimally invasive technique for hemihepatectomy procedures in recent years [20].

Laparoscopic method in pancreatic conditions

Pancreatitis

In practice, acute pancreatitis cases, including its etiology and consequences, are the only pancreatic illnesses that demand an immediate surgical approach, with the exception of injuries to the pancreas treated as abdominal trauma. Admission via index for patients with mild pancreatitis, laparoscopic cholecystectomy is recommended [21]. Open necrosectomy has been linked to substantial death rates (11%-39%) and a high prevalence of complications (34%-95%). As a result, minimally invasive procedures like endoscopic transluminal drainage, percutaneous drainage, and minimally invasive necrosectomy have become more popular in recent years [22]. When compared to open necrosectomy, the step-up method, which begins with percutaneous or endoscopic draining and ends with minimally invasive necrosectomy, has been demonstrated to lower mortality, and complications [23]. Two minimally invasive procedures, video-assisted retroperitoneal debridement (VARD) and minimal access retroperitoneal pancreatic necrosectomy (MARPN), are now commonly used as treatment alternatives [24]. 

*Laparoscopic Cystogastrostomy* 

Using laparoscopic methods, a fistula is made for removing necrotic tissue and draining it. Transgastric, intragastric, or endoluminal laparoscopic cystogastrostomy is possible [4]. 

*Retroperitoneal* *Laparoscopic Debridement*

Severe acute pancreatitis may require retroperitoneal laparoscopic debridement and draining of infected necrosis by the flanks, depending on the location of the necrosis and/or collections. Two or three drains are still present inside the space after the necrotic tissue has been removed [4]. 

Pancreatic Malignancies

Contrary to most other abdominal operations, open surgery is still frequently used to remove malignant tumors from the pancreas. Laparoscopic surgery is safe and may be advantageous in the early postoperative period, according to recently published randomized trials. Similarly, preliminary data on robot-assisted pancreatic excision indicates improvements [25].

Laparoscopic method in small intestine conditions

Acute Small Intestinal Obstruction

A common surgical emergency called small bowel obstruction (SBO) is frequently caused by postoperative adhesions. The majority of patients with adhesive SBO recover without surgery, although a significant number of individuals require urgent surgery. The preferred method for treating SBO has been open surgery for a long period. Laparoscopy is now used in many elective conditions as a treatment of choice, and it is also showing promise as a treatment for this problem [26]. After unsuccessful conservative treatment, open surgery is still the preferred surgical choice for treating strangulated acute small bowel obstruction (ASBO). Nevertheless, laparoscopy with an open-access method through the upper quadrant from the left can be a safe and effective strategy in certain patient groups. Preferably, patients with a first episode of ASBO and/or a single adhesive band should try laparoscopic adhesiolysis. We should keep a low threshold for the open-access method because there may be issues with the application of this technique [27]. There are several potential benefits of laparoscopic adhesiolysis for small bowel obstruction, including enhanced visualization of the abdominal cavity, quicker recovery, early return to full activity, shorter hospital stay, fewer complications, and less chance of adhesion formation [28]. 

*Meckel’s Diverticulum* 

The resection method is used to treat symptomatic Meckel's diverticulum. Laparoscopic diverticulectomy is safe and successful like open surgery, and can speed up the period between oral intake and patient discharge, even if more than 75% of resections are still done that way [29]. To compensate for the lack of sensory feedback, a trans-umbilical technique can be employed. This technique offers the visual and surgical benefits of laparoscopy [30].

Laparoscopic method for conditions of colon and rectum

Since the 1991 description of the first laparoscopic colectomy, it has also been utilized to treat benign and inflammatory disorders in addition to colorectal cancer. Similar to other laparoscopic surgeries, the advantages of laparoscopic colectomy include reduced postoperative pain, a shorter hospital stay, and an earlier return to regular activity [31]. Even though colorectal laparoscopic surgery for elective purposes is a well-established procedure [32].

Appendicitis

The colorectal condition that is probably treated by laparoscopy most frequently in emergency situations is acute appendicitis. Laparoscopic appendectomy takes longer to complete but results in fewer complications, a quick return to normal activities, and higher patient satisfaction [33]. Particularly in cases of appendicitis with perforation, the laparoscopic approach is linked to a shorter hospital stay and a lower incidence of postoperative complications in pediatric patients [34]. Kurt Semm, a gynecologist, performed the first laparoscopic appendicectomy on September 13, 1980, spurred by advancements in gynecological diagnostic laparoscopy [35]. A four-port procedure in which the mesoappendix was ligated by an extracorporeally thrown knot after the appendix was revealed by tying a Roeder knot around its tip. The mesoappendix was then separated from the appendix, and the base was then wrapped with two Roeder loops. After that, the base was split, and the stump was invaginated using a purse-string suture that was applied laparoscopically, followed by a Z-stitch [36].

*Inflammatory Bowel Disease* 

Surgery is more likely to be necessary for patients with inflammatory bowel disease (IBD) at some time in their lives. In recent years, the laparoscopic method of treating this illness has become more popular. A minimally invasive method has been shown to result in longer operating times but shorter hospital stays in cases of intestinal obstruction [37]. For surgical patients with inflammatory bowel disease (IBD), a single-incision laparoscopic technique (SILS) is becoming increasingly popular right now and appealing. The SILS technique, which additionally minimizes MIS, makes only one incision and uses an operating platform that accommodates many working equipment in addition to the laparoscopic camera. Because SILS does not require as many 5- to 12-mm incisions in the abdominal wall as multiport laparoscopy, it provides better cosmetic results [38]. In the past 20 years, the prevalence of pediatric inflammatory bowel disease (PIBD) has increased significantly. Diagnostic laparoscopy was initially described in the middle of the 1970s, but it wasn't widely used in pediatric surgery until the late 1990s. Initially, MIS was only performed for diagnostic reasons. After 2002, it was also used for the aggressive treatment of PIBD, including either ulcerative colitis or Crohn's disease (CD). The "gold standard" for treating PIBD has recently been minimally invasive techniques, which have been favored for the past ten years [39].

*Colorectal Carcinoma* 

**Figure 2:**
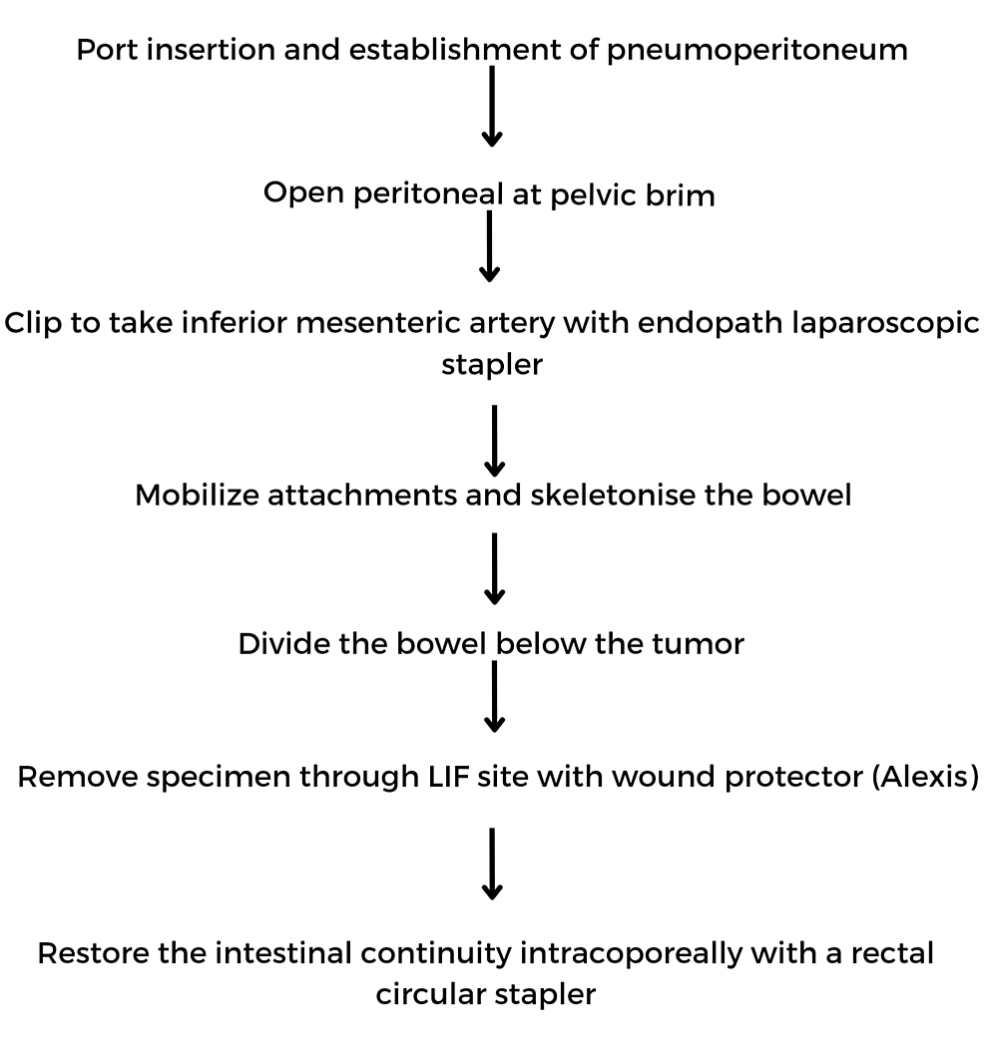
Procedure flowchart of laparoscopic hemicolectomy. Note: This image is the author's own creation.

Over the past 20 years, laparoscopic procedures have been widely used for the surgical management of colorectal cancer. Increasing evidence has shown that, when compared to open surgery, laparoscopic colectomy is associated with immediate and comparable oncologic results [40]. Figure 2 depicts the procedure for laparoscopic hemicolectomy. As the third decade of MIS comes to an end, its acceptance for rectal cancer surgery is rising. As long as the predetermined objectives are achieved, there is insufficient evidence to rank any of the surgical modalities for performing an oncological resection as being superior to the others. (1) Whether or not to undertake an oncological resection vs a local excision by any means; and (2) the choice, tailoring, and timing of non-surgical therapeutic modalities appear to have more influence on results than the choice of technique [41].

Acute Diverticulitis

There are two types of complicated and uncomplicated presentations of acute diverticulitis. Diverticulitis of the complicated is defined as having a fistula, stricture, localized or distant abscess, localized or generalized perforation, or blockage. Laparoscopic lavage has gained recognition as a cutting-edge management technique for complicated diverticulitis, particularly Hinchey III (purulent) diverticulitis since it was first developed in the late 1990s [42].

**Table 2:** Summary of the studies included in the review. MIS: minimally invasive surgery, IBD: inflammatory bowel disease, PIBD: pediatric inflammatory bowel disease. Note: This table is the author's own creation.

Author	Year	Interpretation
Lunca et al. [1]	2005	Operative surgery has been revolutionized by minimally invasive procedures, which are now utilized in the majority of surgical subspecialties.
Jimenez Rodriguez et al. [4]	2016	Laparoscopic surgeries are used in various elective and emergency procedures.
Ben-David et al. [9]	2014	Endoscopic surgery for the placement of esophageal stents with minimally invasive repair is proven to be effective.
Bertleff et al. [10]	2010	Laparoscopy should be used as a diagnostic and therapeutic tool in perforated peptic ulcers.
Suda et al. [12]	2016	In an effort to enhance postoperative outcomes, MIS has been utilized more frequently for upper GI malignancies.
Dziodzio et al. [13]	2021	The surgical procedure of choice for esophageal cancer is increasingly being minimally invasive esophagectomy.
Khan et al. [18]	2008	Age fifty-five years, an abscess of approximately five cm, involving both the lobes of the liver, and an illness lasting for seven days or more are the indications for aspiration for a hepatic abscess.
Cho et al. [20]	2020	Minimally invasive hepatectomy is yet to be standardized as a procedure and hence is limited to expert centers.
Karakayali [22]	2014	Minimally invasive strategies are studied pertaining to the high mortality and morbidity rates in open necrosectomy.
Jensen et al. [25]	2020	Laparoscopic surgery is safe and may be advantageous in the early postoperative period, according to recently published randomized trials.
Di Saverio et al. [27]	2013	With experienced laparoscopic surgeons and a particular group of patients, a laparoscopic approach in acute small bowel obstruction is safe.
Pepper et al. [29]	2012	Laparoscopic diverticulectomy is safe and successful like open surgery, and can speed up the period between oral intake and patient discharge.
Lacy et al. [31]	2002	Laparoscopic colectomy is effective in terms of postoperative pain, a shorter hospital stay, morbidity, and survival.
Kouhia et al. [33]	2010	Laparoscopic appendectomy results in less number of complications, a quick return to normal activities, and higher patient satisfaction.
Nash et al. [37]	2010	A minimally invasive colectomy is safe during emergencies of large bowel conditions.
Holder-Murray et al. [38]	2015	Due to effective short- as well as long-term outcomes MIS performed by experienced surgeons is becoming a standard procedure for IBD.
Pini Prato et al. [39]	2015	The "gold standard" for treating PIBD has recently been minimally invasive techniques.
Zeng et al. [40]	2014	Laparoscopic colectomy is associated with immediate and comparable oncologic results.
Melstrom et al. [41]	2020	As the third decade of MIS comes to an end, its acceptance for rectal cancer surgery is rising.
Hawkins et al. [42]	2020	Laparoscopic lavage has gained recognition as a management technique for complicated diverticulitis.

## Conclusions

In conclusion, with the advancing era of technology and its research in the medical field, minimally invasive surgeries are becoming the surgery of choice and convenient, even from the patient’s point of view. With a learning curve to it, there are a few drawbacks as well. But all said and done, the benefits are more, and hence it is widely acceptable. It's all being used as a first-line treatment in various elective and emergency conditions. Randomized trials on open versus laparoscopic appendicectomy and laparoscopy-assisted colectomy versus open colectomy for treatment of non-metastatic colon cancer, along with a systemic review and meta-analysis on laparoscopic versus open adhesiolysis in patients with adhesive small bowel obstruction, give evidence of the same. The article addresses all the gastrointestinal conditions in which a minimally invasive approach is used and is proving to be effective and convenient.
